# Factors Affecting Patient Satisfaction With Emergency Department Care: An Italian Rural Hospital

**DOI:** 10.5539/gjhs.v7n4p30

**Published:** 2014-12-16

**Authors:** Gabriele Messina, Francesco Vencia, Silvana Mecheroni, Susanna Dionisi, Lorenzo Baragatti, Nicola Nante

**Affiliations:** 1 Department of Molecular and Developmental Medicine, University of Siena, Italy; 2 Postgraduate School of Public Health, University of Siena, Italy; 3 Emergency Medicine and Surgery Unit, Orbetello Hospital, Grosseto, Italy; 4 Local Health Authority 9 of Tuscany Region, Nursing Staff, Grosseto, Italy

**Keywords:** emergency department, nursing care, patient satisfaction, questionnaire, rural hospital

## Abstract

**Background:**

In the emergency department satisfaction is strictly linked to the role of the nurses, namely the first interface between patients and hospital services.

**Objectives:**

The purpose of the study was to identify areas of emergency nursing activity associated with minor or major patient satisfaction.

**Methods:**

A descriptive cross-sectional study was conducted from December 2010 - May 2011, in the rural hospital of Orbetello, Tuscany (Italy). Convenience sampling was used to select patients, namely patients presenting at the emergency unit in the study period. The Consumer Emergency Care Satisfaction Scale was used to collect information on two structured subscale (Caring and Teaching).

**Results:**

259 questionnaire were collected. Analysis indicated that only two characteristics significantly influenced overall satisfaction: “receiving continuous information from personnel about delay” positively effect (OR=7.98; p=0.022) while “waiting time for examination” had a negative effect (OR 0.42; p=0.026)

**Conclusions:**

The study was the first conduced in Italy using this instrument that enabled to obtain much important information about patient satisfaction with nursing care received in the emergency department. The results showing improvements must be related to educational aspects, such as explaining patients the colour waiting list, and communication towards patients, such as informing about emergences that cause queue.

## 1. Introduction

### 1.1 Background

Patient satisfaction with nursing care is considered the major predictive factor of overall satisfaction with hospital care (You et al., 2013) and is a measure of nursing quality often used in studies aimed at demonstrating the benefits of changes in nursing care (Aiken, Sloane, Bruyneel, Van den Heede, & Sermeus, 2013; Coetzee, Klopper, Ellis, & Aiken, 2013). In the emergency department (ED), satisfaction is linked to the role of nurses, namely the first interface between patients and hospital services. The reception of patients is determinant in itself, as well as for the relationship between doctors or nurses and patients (Wysong & Driver, 2009; Oflaz & Vural, 2010). The triage is the first point of contact for most patients coming to the ED. Prompt service is perceived as very important for patients, probably the primary factor determining their judgment (Lee & Yom, 2007). Positive or negative perception of nursing triage can have an impact on patient satisfaction and determine whether or not he will return to a specific ED (Lee & Yom, 2007; Elder et al., 2004).

Most studies have explored the determinants of satisfaction, often combining questionnaires with collection of personal data to assess what consumer characteristics were linked to satisfaction (age, gender, ethnic group, income, education) (Wright et al., 2013). Most researchers also measured overall satisfaction, which includes assessment of care received and impressions of the hospital experience, with special reference to interpersonal relations with the ED nurse or the doctor on duty (Wright et al., 2013; Kupfer & Bond, 2012).

A review of the recent scientific literature shows that most empirical descriptive researches were conducted in the USA, Canada and Asia (Brown, Sandoval, Levinton, & Blackstien-Hirsch, 2005; Senarat & Gunawardena, 2011; Yarris et al., 2012; Sun, Adams, & Burstin, 2001) using about 20 different tools to acquire data on patient satisfaction and its determinants. For example, some studies demonstrated that higher severity on presentation was correlated with less satisfaction (Sun, Adams, & Burstin, 2001). Young age also showed an inverse relationship with perceived quality (Oflaz & Vural, 2010; Raper, Davis, & Scott, 1999). Other studies showed that information provided by ED nurses and their attention to patient care were positive predictors of high overall satisfaction (Parra Hidalgo et al., 2012; Ak et al., 2011; Davis & Duffy, 1999).

In Italy, there have been few studies on patient satisfaction in ED (Alvaro, Brancato, Dessy, & Cammarano, 2003; Iapichino, Vegni, Frangi, Bernasconi, & Moja, 2006; Seghieri, Sandoval, Brown, & Nuti, 2009; Ruggeri et al., 2006) and none on patient satisfaction with the nursing care received.

### 1.2 Aim

The general aim of the research was to measure and study the relationship between patient satisfaction in an ED nursing care using a validated tool, the Consumer Emergency Care Satisfaction Scale (CECSS). Specific aims were: i) to identify areas of nursing activity associated with minor or major patient satisfaction in order to plan future improvements; ii) to identify overall satisfaction of users for nursing care received in the ED and how it is influenced by individual characteristics and examination; iii) to compare the overall satisfaction rating with that of other CECSS-based studies in the literature.

## 2. Methods

### 2.1 Setting

This study is a cross-sectional survey using validated questionnaire. It was conducted in the rural hospital of Orbetello in Grosseto Province, Tuscany, Italy. The Hospital has 86 beds in the following Units: Surgery, Medicine, Emergency Medicine and Surgery, Anesthesia and Intensive Care, Pediatrics, Dialysis and Specialist Clinics. The research project was approved by the hospital ethics committee. The study period was from 1st December 2010 to 31st May 2011.

### 2.2 Sampling

Sample selection was based on a non-probability method, namely patients presenting at the ED in the study period. In particular this was a purposive sampling which can be very useful in situation where the researcher needs to reach a targeted sample quickly and where sampling for proportionality is not the primary concern. With a purposive sample, it is possible to get the opinions of a target population (Smith, 1983; Neyman, 1934). Our study has a specific predefined group to seek. In fact inclusion factors were: age over 18 years, Italian language fluency and literacy, absence of mental disorders, absence of protected custody, and green, white or blue triage color code.

A questionnaire was initially administered to a small sample of 20 patients to verify whether respondents interpreted the questions correctly and were free of comprehension problems (pilot study). Minimal adjustments were made and these scores were not included in the final database.

The choice of focusing on patients with lower colour codes (representing lower urgency) was dictated by the definition of the red and yellow codes, which automatically excluded the possibility of administering a questionnaire (Rancaño-García I, Mar 2013). With regard to benchmarking the results, the Australasian Triage Scale (ATS), the Canadian Triage and Acuity Scale (CTAS), the Manchester Triage System (MTS) and the Emergency Severity Index (ESI) have all proven to be reliable and comparable for assessment of severity by emergency personnel. The triage model used in the study is similar to the Canadian and Australian scales that refers to a five-point scale from 1 to 5 (numeric code) to identify urgency or deferability: red color code=1; yellow color code=2; green color code=3; blue color code=4; white color code=5 (Storm-Versloot, Ubbink, China Choi, & Luitse, 2009).

### 2.3 Data Collection

Data were collected using the Consumer Emergency Care Satisfaction Scale (CECSS), a validated tool based on the literature on patient satisfaction with emergency department (Risser NL, Jan-Feb 1975) and personal experience of the authors in emergency nursing. In the past, the construct validity of the CECSS was verified using the factor analysis performed on aggregate data from various studies (Elder et al., 2004; Raper, Davis, & Scott, 1999; Clark, Pokorny, & Brown, 1996). This led to the elaboration of a 19-item questionnaire used by Davis & Duffy, and Chan & Chau (Davis & Duffy, 1999; Chan & Chau, 2005). This latest version of CECSS instrument was chosen to perform the study.

The CECSS items concern the behaviour, acts and attitudes of nurses towards emergency patients. They are divided into two subscales: a Caring scale (CS) with 12 items (1, 2, 3, 8, 10, 11, 12, 13, 15, 16, 18, 19), and a Teaching scale (TS) with three items (4, 6, 7). Four items (5, 9, 14, 17) are statements in the negative used to increase the reliability of answers but not included in the point score. Answers are scored from 1 to 5, indicating different degrees of agreement or disagreement with the statement: 1 (complete agreement), 2 (substantial agreement), 3 (partial agreement), 4 (slight disagreement), 5 (complete disagreement).

CECSS was then translated into Italian by an independent translator. The technical team corrected any formulations that did not match the particular situation. The resulting text was translated back into English by another translator in order to check whether the translation was the same as the original without changes in meaning.

CECSS was complemented by a personal details form including: age, gender, nationality, marital status, education, pain, previous hospitalisation, waiting time before and after examination, color code assigned at triage, information received, nursing intervention, and overall satisfaction.

### 2.4 Ethical Consideration

The study did not require the Institutional Reviewed Board:

- all available data were completely anonymous;
- the study was not limited to course-related activities designed specifically for educational or teaching purposes;
- no identifiable private information were obtained about living individuals;
- the data or specimens received by or provided did not contain identifiable private information;
- the data or specimens were not coded and there was not a link that would allow the data or samples to be identified.

Nevertheless the project was implemented with the authorization and support of the hospital medical direction. Administration of the questionnaire observed all national laws on patient information, anonymity and privacy.

The questionnaire was accompanied by an informative letter, which described the project and purpose. All participants were asked to sign an informed consent before where they were given explanations on how to take part to the project.

Previously a signed permission was obtained from Barbara A. Davis (who validated the CECCS instrument) to use the CECCS in the research.

The ED personnel included a nursing team (6 emergency nurses), support personnel (two registered nurses, one practical nurse, three technicians) and reception personnel (two practical nurses and one technician). The ED personnel who distributed the questionnaires had attended a 1-week preparation course. The nursing manager facilitated the implementation of the survey and designated a research nurse to supervise the data collection.

The patients completed the questionnaires anonymously and returned them in a sealed envelope to a sealed box at the ED. These strategies endeavoured to protect the privacy of patient and increase the response rate. Subsequently the nurse manager returned the sealed questionnaires to the research nurse who returned all sealed questionnaires to the study director from the university.

### 2.5 Data Analysis

All questionnaires in the study period were processed using optical-recognition software Remark Office version 7.0 (Gravic Inc., Malvern, PA, USA), that enabled standardisation of the method of reading the answers.

First was performed a descriptive analysis of demographic data and emergency examination. The percentages of answers to the CECSS items were calculated to establish the strong and weak points of emergency service care. To account for possible selection bias between respondents and non-respondents to all CECCS items, a χ^2^ test was performed for statistical significance. Then was conducted a bivariate analysis of the independent variables (e.g. age, gender, marital status) and the dependent variable (overall satisfaction – OS). Statistically significant variables were then entered in a logistic regression model. Significance was set at P<0.05. Stata ® SE version 12.1 (StataCorp, College Station, Texas, USA) software was used for the analysis.

The Instrument reliability was evaluated through the internal consistency of each dimension (teaching and caring) and of the whole scale. In the Spanish validation (Cunado, Garcia, Rial, & Garcia, 2002), the Cronbach alpha coefficient values were greater than 0.8, consistent with the values shown by other studies (Davis & Duffy, 1999).

Validity was assured by using lines of questioning to verify accuracy and consistency of Italian translation. A team of nursing experts of the hospital confirmed that the instrument was valid and unambiguous.

## 3. Results

The number of questionnaires collected was 259. The χ^2^ tests did not highlight any possible bias comparing respondents and non-respondents for the variables/items included in CS (p=0.275) and gave statistical significant value only for the TS (p=0.026). The latter was not significantly associated with OS at the bivariate analysis, consequently it was ruled out from the regression model and did not represent a possible source of bias in the final analysis.

At a descriptive analysis dichotomous results were obtained for the various items of the CECSS. The items typical of the Caring scale were the ones most often judged positively: for example 255 (98.5%) patients answered that nurses performed their duties with skill. On the other hand, many of the items investigating nurses’ instructions to patients at discharge or dedicating time to patient needs, typical of the Teaching scale, were judged more severely: for example 113 (43.6%) patients -answered that nurses did not give sufficient instructions and that they did not inform the patient what to expect at home. The question on overall satisfaction received very high score in the whole study period, a total of 254 (98.0%) patients declared that they were satisfied or very satisfied. The results are summarised in Table 1.

**Table 1:** Percentage distribution of the CECCS items answered and overall satisfaction (n 259)

Statement (item)	n (%)					

	Total disagreement			Total agreement		*Missing*

	1	2	3	4	5
1. The nurse performed his/her duties with skill	0 (0.0)	1 (0.4)	1 (0.4)	2 (0.8)	255 (98.5)	0 (0.0)
2. The nurse seemed to know something about my illness/problem	17 (6.6)	0 (0.0)	15 (5.8)	5 (1.9)	215 (83)	7 (2.7)
3. The nurse knew what treatment I needed	21 (8.1)	3 (1.2)	19 (7.3)	6 (2.3)	199 (76.8)	11 (4.2)
4. The nurse gave me instructions about caring for myself at home	113 (43.6)	0 (0.0)	6 (2.3)	1 (0.4)	130 (50.2)	9 (3.5)
5. The nurse should have paid more attention to what it was	238 (91.9)	1 (0.4)	5 (1.9)	1 (0.4)	9 (3.5)	5 (1.9)
6. The nurse told me what problems to watch for	77 (29.7)	2 (0.8)	2 (0.8)	3 (1.2)	162 (62.5)	13 (5.0)
7. The nurse told me what to expect at home	117 (45.2)	0 (0.0)	1 (0.4)	3 (1.2)	125 (48.3)	13 (5.0)
8. The nurse explained all procedures before they were done	20 (7.7)	1 (0.4)	4 (1.5)	2 (0.8)	219 (84.6)	13 (5.0)
9. The nurse seemed too busy controlling the sickbay to dedicate time to speak to me	236 (91.1)	0 (0.0)	6 (2.3)	0 (0.0)	9 (3.5)	8 (3.1)
10. The nurse explained things in terms I could understand	5 (1.9)	1 (0.4)	6 (2.3)	2 (0.8)	240 (92.7)	5 (1.9)
11. The nurse was understanding when listening to my problem	2 (0.8)	1 (0.4)	10 (3.9)	4 (1.5)	227 (87.6)	15 (5.8)
12. The nurse seemed genuinely concerned about my pain, fear and anxiety	7 (2.7)	0 (0.0)	9 (3.5)	7 (2.7)	207 (79.9)	29 (11.2)
13. The nurse was as gentle as he/she could be when performing painful procedures	3 (1.2)	2 (0.8)	3 (1.2)	4 (1.5)	226 (87.3)	21 (8.1)
14. The nurse treated me more like a number than a person	240 (92.7)	1 (0.4)	2 (0.8)	3 (1.2)	10 (3.9)	3 (1.2)
15. Seemed to understand how I felt	7 (2.7)	0 (0.0)	14 (5.4)	4 (1.5)	223 (86.1)	11 (4.2)
16. The nurse gave me a chance to ask questions	10 (3.9)	1 (0.4)	5 (1.9)	3 (1.2)	225 (86.9)	15 (5.8)
17. The nurse was not kind	251 (96.9)	3 (1.2)	0 (0.0)	0 (0.0)	3 (1.2)	2 (0.8)
18. The nurse appeared to take time to meet my needs	96 (37.1)	1 (0.4)	44 (17.0)	5 (1.9)	102 (39.4)	11 (4.2)
19. The nurse made sure all my questions were answered	11 (4.2)	2 (0.8)	5 (1.9)	1 (0.4)	224 (86.5)	16 (6.2)

		Very Poor	Poor	Satisfied	Very satisfied	*Missing*
Overall, how do you rate your satisfaction with ED service?		0 (0.0)	5 (2.0)	171 (66)	83 (32)	0 (0.0)

Table 1 describes the percentage of distribution of the answers to CECCS items and overall satisfaction. In the first column are listed in order the nineteen items of the questionnaire, the last one (twentieth) is overall satisfaction. In the subsequent 5 columns there are the 5 possible degrees of the likert scale (1= total disagreement; 2= partial disagreement; 3= neutral; 4= partial agreement; 5 total agreement) with the percentage registered for each items; the last one is a 4 point likert scale referring to the overall satisfaction with the hospital experience.

The study also investigated the effect of independent variables as patient characteristics and their experience with Emergency Room admission, on overall satisfaction (dependent variable), describing the strength of this association with the Odds Ratio (OR) from the regression analysis. Bivariate analysis indicated that only two features significantly influenced the score of overall satisfaction. Receiving continuous information from personnel about delayed medical examination had a positive effect on OS (OR=7.98, p=0.022) with respect to not receiving such information. Waiting time for examination between 30-60 minutes instead of the gold standard of < 30 minutes, had a negative effect on OS (OR 0.42, p=0.026). It seems important to mention also that higher levels of patient education seemed associated with OS, showing OR=2.49 and borderline significance p=0.056. Besides demographic details and waiting time for examination, the research also investigated whether OS was associated with the Caring scale and Teaching scale scores. Such influence was only found for the Caring scale: those giving a high Caring score had a significantly higher probability of good OS (OR 7.68, p= 0.025) (Table 2a-b).

**Table 2:** Bivariate analysis of emergency patient characteristics in relation to overall perceived satisfaction (outcome)

Exposure variable	Respondents	Overall Satisfaction		
	n (%)	OR	95% CI	*P*
**Age**				
	18 - 29 y	38 (14.6)	1	─
30 - 39 y	30 (11.5)	0.45	0.14 - 1.45	0.168
40 - 49 y	54 (20.9)	1.41	0.60 - 3.28	0.428
50 - 59 y	40 (15.7)	0.79	0.31 - 2.02	0.616
60 - 69 y	36 (14.1)	1.53	0.61 - 3.87	0.365
> 69 y	61 (23.2)	0.93	0.40 - 2.20	0.876
Missing	0 (0.0)	─	─	─
**Gender**				
Male	142 (45.1)	1	─	─
Female	117 (54.9)	1.04	0.61 - 1.75	0.893
Missing	0 (0.0)	─	─	─
**Marital status**				
Unmarried	63 (24.5)	1	─	─
Married	147 (56.8)	1.19	0.62 - 2.30	0.605
Divorced	49 (18.7)	0.73	0.30 - 1.78	0.497
Missing	0 (0.0)	─	─	─
**Time of discharge**				
> 30 minutes after admission	97 (36.9)	1	─	─
1-3 hours after admission	132 (51.4)	0.73	0.42 - 1.27	0.265
3-6 hours after admission	25 (9.7)	0.36	0.13 - 1.08	0.058
> 6 hours after admission	2 (0.8)			
Missing	3 (1.2)	─	─	─
**Information about delays**				
No information from personnel about delays	18 (7.2)	1	─	─
Little Information from personnel about delays	27 (10.5)	3.86	0.39 - 38.7	0.215
Continuous information from personnel about delays	97 (36.9)	7.98	0.96 - 66.50	0.022
Not applicable	115 (44.5)	─	─	─
Missing	1 (0.4)	─	─	─
**Coulor Code**				
Blue (weak category)	37 (14.6)	1	─	─
White (not urgent)	18 (7)	0.75	0.14 - 3.93	0.73
Green (postponable)	204 (78.4)	1.57	0.40 - 6.12	0.51
Missing	0 (0.0)	─	─	─
**Educational qualification**				
Primary	66 (25.5)	1	─	─
Lower	79 (30.5)	0.89	0.43 - 1.83	0.746
Upper Secondary	89 (34.0)	1.02	0.51 - 2.04	0.959
Tertiary	25 (9.6)	2.49	0.95 - 6.57	0.056
Missing	1 (0.4)	─	─	─
**Pain**				
Absence of pain	49 (19.1)	1	─	─
Mild pain	57 (21.9)	1.06	0.46 - 2.47	0.888
Moderate pain	81 (31.2)	1.18	0.54 - 2.58	0.674
Severe pain	70 (27.1)	1.39	0.63 - 3.08	0.416
Missing	2 (0.8)	─	─	─
**Previous hospitalization**				
No previous hospitalization	162 (62.8)	1	─	─
1 previous hospitalization	34 (12.8)	0.83	0.37 - 1.87	0.658
2 previous hospitalizations	24 (9.2)	0.53	0.19 - 1.50	0.221
≥ 3 previous hospitalizations	36 (14)	1.13	0.53 - 2.41	0.751
Missing	3 (1.2)	─	─	─
**Waiting time until medical examination**				
≥ 30 minutes	187 (72.3)	1	─	─
30-60 minutes	46 (17.7)	0.42	0.19 - 0.92	0.026
1-2 hours	24 (9.2)	0.41	0.15 - 1.14	0.077
≥ 3 hours	2 (0.8)			
Missing	0 (0.0)	─	─	─
**Caring Scale (CS)**				
Not satisfied (12–32)	13 (5.0)	1	─	─
Neutral (33-42)	38 (14.7)	3.72	0.40 - 34.60	0.214
Satisfied (43-60)	146 (56.4)	7.68	0.93 - 63.22	0.025
Missing	62 (23.9)	─	─	─
**Teaching Scale (TS)**				
Not satisfied (3–7)	109 (42.1)	1	─	─
Neutral (8-10)	22 (8.5)	0.35	0.09 - 1.28	0.096
Satisfied (11-15)	107 (41.3)	1.54	0.88 - 2.71	0.129
Missing	21 (8.1)	─	─	─

Table 2 (a-b) contains a bivariate analysis conducted between independent variables (patient characteristic, the experience with Emergency Room admission, Caring Scale and Teaching Scale) and the dependent variable (Overall Satisfaction). In the first column are listed the exposure variables (independent) and their possible range/level. In the second column are reported the percentages recorded for each range/level. In the subsequent 3 columns are reported, respectively: the Odds Ratio obtained for each range/level (the first range/level of each variable is taken as base line in the comparison with the other, so is reported as OR=1); the 95% Confidence Interval (the first range/level of each variable, control base line, had no 95% CI); the P value (in order to establish if the relationship between exposure variables and Overall satisfaction is statistically significant).

When the variables that affected bivariate analysis were put into the logistic regression model, no significant effect on OS was found.

## 4. Discussion

Some important aspects emerge from the present study: 1) with regard to method, the questionnaire developed for English-speaking countries and adapted by us for Italy, proved practical and well-understood by patients; 2) consistent with other studies on ED nursing care (Chan & Chau, 2005; Clark, Pokorny, & Brown, 1996; Wysong & Driver, 2009) the items obtaining the highest score regarded care received, whereas the lowest scores were for the teaching function of nurses; 3) only two items were associated in a significant way with OS, but only according to bivariate analysis. As already mentioned, this aspect of the study was complicated by the fact that almost all patients expressed a positive or very positive judgment. The aim of the study was therefore limited to examining this fine difference in OS. With regard to the possible association of OS with the various items of the subscales, only the Caring subscale was a predictor of better OS according to bivariate analysis. The Teaching subscale was considerably less associated with OS and not significantly. With regard to access to emergency medical care, only waiting time (satisfaction decreased as waiting time increased) and information received about any delay (satisfaction increased when the nurse provided explanations of delay) determined patient satisfaction. Other studies showed that information provided by triage nurses and their attention to patient care were positive predictors of high overall satisfaction (Oxler, 1997; R, 2001; Perucca, 2001).

Some authors have demonstrated that also variables such as pain and anxiety caused by the emotional stress of the emergency may play a major role in the way patients answer the questionnaire: patients who were severe on admission had less motivation to fill in the questionnaire. In our study this aspect presumably did not play an important role because the colour codes considered were the least severe ones (Boudreaux ED, 2004; Shilling V, 2003).

The study, however, highlighted important areas of nursing care on which to initiate improvements. In fact, with regard to comparison of the Caring and Teaching aspects, the former subscale elicited extremely positive responses in all prior studies based on the same tool (Davis & Duffy, 1999; Cunado et al., 2002; Chan & Chau, 2005), whereas the latter subscale elicited less favourable responses. For example, in the study items with the highest percentage agreement focused on technical abilities, attention of nurses, clear communication and sensitivity during procedures. Items with the greatest disagreement, on the other hand, belonged essentially to the Teaching subscale and revealed critical aspects of information and teaching in relation to discharge of the patient and in the time taken to answer questions about the pathology.

Comparing the mean scores of the two subscales, they were among the lowest in the studies using the same tool (Table 3).

**Table 3:** Comparison of mean Caring and Teaching scale scores (TS and CS) by authors who used the CECCS questionnaire

AUTHOR	CS	TS

	(range 12 - 60)	(range 3 - 15)
Messina et al.	43.46±4.84	6.94±5.21
Chan & Chau	43.93±6.09	10.37±2.81
Cunado et al.	50.50±7.80	10.20±3.50
Davis & Duffy	55±6.49	10.82±2.99
Raper et al.	48.80	/

Table 3 compares the results obtained in the studies found in literature conducted with the same instruments. In particular we focused the attention on the mean scores registered with the two scales (Teaching Scale and Caring Scales) by the authors. In the first column are listed the 5 authors that used the CECCS questionnaire (in the first position we placed our article). In the subsequent two columns are expressed the mean scores (with the possible variance) obtained by each authors respectively in the Caring Scales (considering the answers to items: 1, 2, 3, 8, 10, 11, 12, 13, 15, 16, 18, 19) and the Teaching Scale (considering the answers to items: 4, 6, 7).

An element to consider is that the sample (n=259), being much larger than those examined with the same methodology: the Australian study with 103 patients (Raper, Davis, & Scott, 1999); the Chinese study with 56 patients(Yarris et al., 2012); the Spanish study with 93 patients (You et al., 2013), and being performed by a different method (direct interview versus telephone interview 2 weeks later) from the only study with a larger population (the English study with 378 patients (Oflaz & Vural, 2010), may have been more reliable in detecting certain aspects of dissatisfaction linked to particular items, not discussed in the other studies.

## 5. Limits

With regard to use of CECSS in the Italian context, the questionnaire was quite well received by patients. This was also confirmed by the fact that very few patients asked the ED nurses for help in answering or understanding the questions. However an emerging issue was the dichotomy of replies: patient opinion was often “complete agreement” or “complete disagreement”. No specific explanation of this problem of discriminating shades of meaning in replies could be found in the literature. The more likely reason was that patients gave extreme replies to emphasise their perception of service.

Another limitation was that the direct administration of questionnaires is certainly the most simple and economical way, ensuring the lowest number of missing answers but on the other hand it present some kind of information and selection bias. However the validation studies found CECSS structured to be administered in all the different ways.

## 6. Conclusions

This study, the first conducted in Italy using CECSS, was designed to obtain much important information about patient satisfaction with nursing care received in the emergency department. On the whole, the results were encouraging, showing high levels of overall satisfaction, especially for the clinical aspects of nursing care.

The research highlighted particular deficiencies. Results suggest that the margins for improvement are small for the population enrolled in this study, however some progresses are possible: reorganization of aspects of patient discharge, which is mainly the responsibility of the referring doctor; improvement of the teaching capacity of nurses in communicating with patients, reassuring them about what to do on their return home; more time dedicated to listening to doubts and needs of patients, even if this means less time for clinical care.
